# Structural changes of *Chlamydomonas reinhardtii* cells during lipid enrichment and after solvent exposure

**DOI:** 10.1016/j.dib.2018.02.042

**Published:** 2018-02-15

**Authors:** Sakina Bensalem, Filipa Lopes, Pierre Bodénès, Dominique Pareau, Olivier Français, Bruno Le Pioufle

**Affiliations:** a CNRS, SATIE, Ecole Normale Supérieure Paris Saclay, Université Paris-Saclay, 61 av du Pdt Wilson, 94230 Cachan, France; b LGPM, EA 4038, CentraleSupélec, Université Paris Saclay, 3 Av. Joliot Curie, 91190 Gif-sur-Yvette, France; c ESIEE-Paris, ESYCOM EA 2552, Université Paris Est, 93160 Noisy Le Grand, France

## Abstract

Data are related to Confocal Laser Scanning Microscopy (CLSM) observations of lipid-enriched *Chlamydomonas reinhardtii* cells under different conditions. Firstly, the impact of stress conditions (nitrogen starvation) on the cell wall structure is assessed. Secondly is described the effect of solvents, in the context of lipid extraction, on the microalga's cell, particularly its lipid droplets, in the perspective of understanding the mechanisms behind solvent extraction of lipids. Furthermore, the role of the cell wall as a barrier to the solvent extraction action is highlighted.

**Specifications table**

**Table:** 

Subject area	Physics and biology
More specific subject area	Bioprocess engineering
Type of data	Confocal microscopy images
How data was acquired	Images were acquired by confocal microscope LSM 700 (Zeiss)
Data format	Raw
Experimental factors	*Chlamydomonas reinhardtii* cells were submitted to 7 days of nitrogen stress conditions in order to induce lipid accumulation
Experimental features	Fluorescent dyes were used to observe the microalga’s lipid droplets and cell wall
Data source location	*Chlamydomonas reinhardtii* SAG-34.98 (Wild type) was obtained from the University of Goettingen (EPSAG, Germany). They were collected from a soil in the United States, MA.
Data accessibility	Dataset is within this article
Related research article	Understanding the mechanisms of lipid extraction from microalga *Chlamydomonas reinhardtii* after electrical field solicitations and mechanical stress within a microfluidic device (Bensalem et al., 2018) [1] (In revision)

**Value of the data**

• The presented data provides information on the role of the cell wall as a barrier to molecules extraction; this information is of prime importance for lipid extraction from microalgae
• The data will be helpful to relate the microalga's cell wall structure and the ability of a given solvent to extract lipids
• The data gives significant information that might be crucial to highlight mechanisms of solvent extraction action, especially the role of the cell wall in the extraction efficiency
• The data presented here might lead to further investigations on microalgae cell wall during stress conditions and solvent extraction
• The data provides information to the scientific community on the effect of coupled solvents on the microalgae cells structure in the frame of the extraction of lipids from microalgae

## 1 Data

**Fig. 1:**
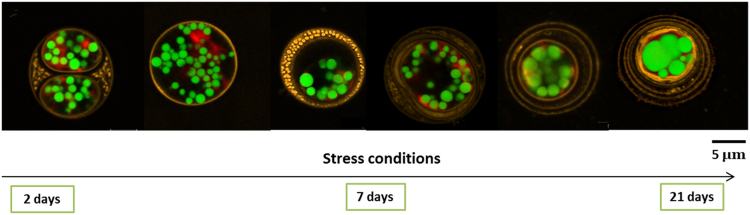
*Chlamydomonas reinhardtii* observed under confocal microscopy during lipid accumulation. Cells evolve during stress conditions in terms of their cell wall. Different profiles could be observed: cells in division, cells with one thin layer of cell wall, cells with one very thick layer of cell wall, 2 to 3 layers of cell wall, and more than 3 layers, up to 7 layers. Lipid droplets were stained with Bodipy 505/515 (green), the cell wall with Concanavalin A (orange). Scale bar : 5 µm.

**Fig. 2:**
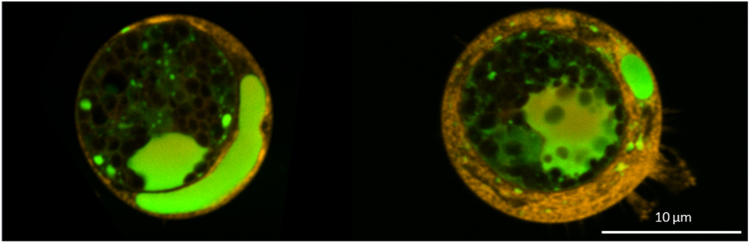
Confocal microscopic observations of 7-days stressed *Chlamydomonas reinhardtii* cells after solvent contact with ethyl acetate. *Chlamydomonas reinhardtii* cells were mixed with ethyl acetate in a total volume of 100 µL during 10 min (7.5 µL of ethyl acetate was added to the algal solution). In green, lipid droplets stained by bodipy 505/515 and in orange the cell wall stained by concanavalin A. Images show the lipid droplets being trapped in the cell wall. Scale bar: 10 µm.

**Fig. 3:**
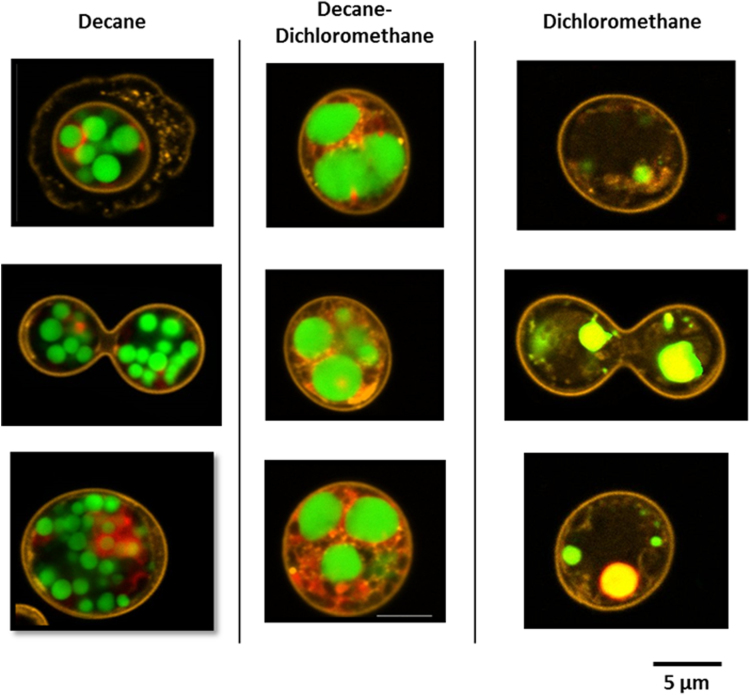
Confocal microscopic observation of 7-days stressed *Chlamydomonas reinhardtii* cells after being in contact with different solvents. The solvent mixture was used with a ratio of 1:1; dodecane as the biocompatible solvent with dichloromethane as the toxic solvent. The solvent/cell ratio was 1:1 and the contact time was 30 min. Lipid droplets were stained with bodipy 505/515 (green), and the cell wall with concanavalin A (orange). Scale bar: 5 µm.

Three experiments using confocal microscopy imaging on 7 days-stressed *Chlamydomonas reinhardtii* are reported. Fig. 1 shows the cytoplasm containing lipid droplets and the cell wall structure, while the microalgae are under nitrogen starvation, which induces lipid accumulation. Fig. 2, Fig. 3 describes the structural changes on lipid droplets on such stressed cells, when using solvents for lipid extraction purposes. The evolution of the 7 layers composing the cell wall of *Chlamydomonas reinhardtii*, during stress conditions, is shown for the first time in Fig. 1. Fig. 2 offers an original observation of the cell wall entrapping lipids after solvent contact, showing therefore the major role of the cell wall as a barrier to solvent extraction.

Fig. 3 shows the structural changes on the cell, when submitted either to pure or mixtures of polar or nonpolar solvents.

## 2 Experimental design, materials, and methods

### 2.1 Stress conditions

Lipid accumulation was induced by culturing the cells under the following conditions: nitrogen depleted TAP medium, light intensity of 150 μmol·m^−2^·s^−1^, 100 rpm agitation, a temperature of 24 °C ± 1 °C and 0.004% CO_2_.

### 2.2 Microscopic experiments

In order to visualize the cell wall and lipid droplets using confocal laser scanning microscope (CLSM 700, Carl Zeiss Microscopy GmbH, Germany) two fluorescent dyes were used: Concanavalin A conjugated with tetramethylrhodamine (ConA, yellow-orange fluorescence) for the cell wall and Bodipy 505/515 (green fluorescence) for the lipids. Chlorophyll was detected by red autofluorescence.

The stock solution of ConA (Molecular Probes, Lifetechnologies, C860) was prepared by dissolving it in 0.1 M sodium bicarbonate (pH ≈ 8.3) to a final concentration of 2 mg/ml. Fluorescence intensity was analyzed after 20 min of incubation time.

Bodipy 505/515 (green fluorescence) was prepared by dissolving it in DMSO to a concentration of 100 mg/L. The final concentration of bodipy in the cell sample was 1.5 µg/ml. Fluorescence intensity was analyzed after 10 min of incubation time.

### 2.3 Solvent extraction

#### 2.3.1 Ethyl acetate

For this experiment, *Chlamydomonas reinhardtii* cells were mixed with ethyl acetate in a total volume of 100 µL during 10 min (7.5 µL of ethyl acetate was added to the algal solution).

#### 2.3.2 Mixture of solvents

The following solvent mixture was used with a ratio of 1:1: dodecane as the biocompatible solvent with dichloromethane as the highly polar solvent. The solvent/cell ratio was 1:1 and the contact time was 30 min. The cell concentration of the sample was 1 × 10^7^ cells·mL^−1^.

## References

[1] Bensalem S., Bodénès P., Pareau D., Lopes F., Français O., Le Pioufle B. (2018). Understanding the mechanisms of lipid extraction from microalga Chlamydomonas reinhardtii after electrical field solicitations and mechanical stress within a microfluidic device. Bioresour. Technol..

